# Broad-complex Z3 contributes to the ecdysone-mediated transcriptional regulation of the *vitellogenin* gene in *Bombus lantschouensis*

**DOI:** 10.1371/journal.pone.0207275

**Published:** 2018-11-15

**Authors:** Congai Zhen, Huipeng Yang, Shudong Luo, Jiaxing Huang, Jie Wu

**Affiliations:** Key Laboratory for Insect-Pollinator Biology of the Ministry of Agriculture, Institute of Apicultural Research, Chinese Academy of Agricultural Sciences, Beijing, PR China; Indiana University, UNITED STATES

## Abstract

During reproduction, vitellogenin (Vg), as an egg yolk precursor, is critical in sexually mature females of oviparous species including some insects. The transcription of *Vg* is usually mediated by hormones such as juvenile hormone (JH), ecdysteroids and some neuropeptides. In this study, the structure of the *Vg* gene from the bumblebee *Bombus lantschouensis*, (*BlVg*) was determined by sequencing and assembly. *BlVg* was found to be expressed at higher levels in reproductive queens than in virgins by quantitative real-time PCR analysis. Tissue-specific expression analysis showed that *BlVg* was expressed at the highest levels in the fat bodies of both virgin and reproductive queens. Prediction of the *BlVg* promoter revealed the presence of ecdysteroid-responsive cis-regulatory elements (CREs) containing one Broad-Complex zinc-finger isoform 3 (BR-C Z3), and one ecdysone-induced protein 74A (E74A). In addition, luciferase reporter expression, driven by the 5' -regulatory region of the *BlVg* gene, from −1517 bp to +895 bp downstream of the start codon, was induced by treatment with 20-hydroxyecdysone (20-E). Moreover, the luciferase activity of the *BlVg* promoter was elevated by only BlBrC-Z3 when *Sf9* cells were cotransfected with four BlBrC isoforms respectively. *BlVg* promoter-mediated luciferase activation was significantly reduced when the putative BrC-Z3 CRE in the promoter was mutated. In summary, this report describes the first study of vitellogenin gene regulation at the transcriptional level in bumblebees and demonstrates that the ecdysone-induced transcription of the *BlVg* gene is mediated by the binding of BlBrC-Z3 to the BrC-Z3 CRE in the *BlVg* promoter in bumblebees.

## Introduction

Insect vitellogenin (Vg), a large phosphoglycolipoprotein, is the major yolk protein precursor synthesized mainly by the fat body in sexually mature females [1, 2]. Because of its association with fecundity and reproduction, Vg has been extensively studied in many insects, including Hymenoptera. In addition to its traditional role as a vitellin precursor in reproduction, Vg is also known to involve in the development of honeybees, such as social behavior/organization [3–6], aging [7–10], and protection from various stress [11, 12]. As pollinator, bumblebees are important for agriculture and ecological systems. Meanwhile, Vg plays a crucial role in insect reproduction. Hence, it is necessary to decipher the transcriptional profiles and regulation mechanism of the *Vg* gene in *Bombus lantschouensis*.

Lockett et al. summarized the transcriptional expression patterns of hymenopterous *Vg* for various ages, castes and reproductive statuses [13]. The expression profiles of *Vg* were elucidated in some bumblebee species, including *Bombus terrestris* [14], *Bombus ignitus* [15, 16], and *Bombus hypocrita* [17]. For the queens and workers of *B*. *ignitus* and *B*. *hypocrita*, the expression of *Vg* was higher in adults than in pupae [15, 17]. Transcripts of *Vg* were also detected in drone pupae and adults [16, 17]. In conclusion, the mRNA levels of *Vg* were distinct in bumblebees of different castes, especially under different reproductive stages.

Transcriptional regulation of the *Vg* gene is regulated by hormones, including juvenile hormone (JH), ecdysteroids and some neuropeptides [2, 18]. In hymenopterans, a high JH titer and abdominal activation of JH signaling may induce neotenic reproduction in *Reticulitermes speratus* [19]. JH was shown to increase *Vg* synthesis in reproductive bumblebee females by simultaneously monitoring the transcripts of methylfarnesoateepoxidase (*MFE*) and *Vg* [14]. There was also some evidence for the role of JH in reproduction, determined via manipulation of JH signaling by JH-I treatment [20] or treatment with allatoxin precocene-I [21], and a combination of allatectomy and JH-III replacement therapy, and the dependence of the ovarian development and *Vg* expression on JH [22]. In contrast, in some cases, *Vg* was uncoupled from or even negatively correlated with JH levels in *Bombus* species [23].

*Vitellogenin* transcription is hormonally regulated by transcription factors interacting with the upstream regulatory region of the *Vg* gene. Binding sites for multiple ecdysone-related transcription factors, such as the ecdysteroid receptor complex (EcR-USP), and the 20E early-response genes Broad-Complex (BrC), E74 and E75, were detected in the upstream region of the mosquito *Aedes aegypti Vg*.

BrC, as a critical member of the early ecdysone genes, is a kind of DNA-binding protein with N-terminal Bric-a-brac-Tramtrack-Broad (BTB) domains and C2H2-type zinc-finger. Four or more alternatively spliced isoforms of BrC (Z1–Z4, Z1–Z6) were identified in the tobacco hornworm *Manduca sexta* [24], honeybee *Apis mellifera* L. [25], German cockroach *Blattella germanica* [26], yellow-spotted longicorn beetle *Psacothea hilaris* [27], and silkworm *Bombyx mori* [28, 29]. In the mosquito *A*. *aegypti*, BrC Z1 and Z4 served as repressors, inhibiting the 20E-mediated activation of the *Vg* promoter via the ecdysone receptor (EcR-USP), while Z2 enhanced this activation [30, 31]. In the silkworm *B*. *mori*, BmBrC-Z2 was shown to regulate ecdysone-mediated *BmVg* transcription by directly binding to the cis-response elements (CREs) on the *BmVg* promoter [32]. Moreover, the POU homeodomain transcription factor BmPOUM2 was also found to regulate *BmVg* expression by collaboratively interacting with only BmBrC-Z2 [33].

To date, little is known about the regulation of *Vg* transcription in bumblebees. In this paper, we describe the gene structure, expression pattern and transcriptional regulation of the *Vg* gene from the bumblebee *B*. *lantschouensis* (*BlVg*). The mRNA expression patterns of *BlVg* were elucidated for various tissues and reproductive stages of the queen. The mechanism by which the transcriptional factor BlBrC-Z3 is involved in the transcriptional regulation of *BlVg* under 20E was elucidated using luciferase reporter expression.

## Materials and methods

### Samples

*B*. *lantschouensis* bumblebees were reared with sugar syrup (50: 50, v:v) and pollen pellets under standard conditions (29 ± 0.5°C temperature; 55 ± 5% relative humidity; continuous darkness) in the laboratory at the Institute of Apicultural Research, Chinese Academy of Agricultural Sciences.

Various tissues (head, flight muscle, fat body, ovary) were dissected from virgin and reproductive queens respectively, frozen in liquid nitrogen, and then stored at -80°C until RNA isolation.

### Isolation of genomic DNA and total RNA

Genomic DNA was extracted with the Wizard Genomic DNA Purification Kit (Promega, USA). Total RNA was prepared using TRIzol reagent (Invitrogen, USA) according to the manufacturer’s instructions. The quality and concentration of the DNA and RNA extracts were assessed with gel electrophoresis and a Micro-Volume UV-VIS spectrophotometer (NanoDrop-2000, USA). First-strand cDNA was synthesized using PrimeScript RT Reagent Kit with gDNA Eraser (Takara, China). The cDNA was stored at -20°C until further use.

### Cloning of putative *BlVg* and *BlBrC* isoforms

PCR amplification was carried out using cDNA and genomic DNA as templates. Primers used for amplification of the putative *BlVg* and *BlBrC* isoforms (Z1–Z4) were designed based on the *Vg* sequence of *B*. *hypocrita* (GenBank accession no. GQ340749) and the *BrC* isoform mRNA sequence of *B*. *terrestris* (GenBank accession no. NM_001280921) (Table 1). The PCR conditions were as follows: 94°C for 2 min; 30 cycles of 94°C for 30 s, 55°C for 30 s, and 72°C for 2 min; and a final extension period of 72°C for 10 min. The PCR product was subcloned into a pEASY-blunt cloning vector (TransGen Biotech, China) and sequenced with a 3730XL sequencer (ABI, USA).

**Table 1:** Primers used in this study. Restriction enzyme sites are shown in red color and underlined.

Primer name	5’-3’ sequence	Target	Amplicon (bp)
Vg-1-FP	TGATGTCCACGGGTTTCAAC	*BlVg* CDS/ gene	1466/3110
Vg-1-RP	GTCTTGTCCATCGATTTCAG		
Vg-2-FP	TCGATAAGCACGTACGTCTC	*BlVg* CDS/ gene	1530/1860
Vg-2-RP	AGCTCATTCTTCCTTGAATC		
Vg-3-FP	GACGCGAAGAATCTAAAGAC	*BlVg* CDS/ gene	1270/1270
Vg-3-RP	CGACCTTAAGATCCGATAAC		
Vg-4-FP	TCCAATCGCAACATTTCATC	*BlVg* CDS/ gene	1301/1931
Vg-4-RP	TTAGGCCTTGCAAGCAAGAG		
Pro-first -RP	AGGATTTTCGCAGATTTACGATACAC	*BlVg* promoter	2418
Pro-second -RP	TAGAACTGAATTTTCATTGTCTCGAG		
Pro-third -RP	AGAAGATCGATGAGAAAGAAGATG		
Pro-Fp-1	CGGCTAGCATGTTTTTAGAAGTACGAGC	*BlVg* promoter	2418
Pro-Fp-2	CGGCTAGCCGATTAATTTAAGTTGAACC	*BlVg* promoter	2297
Pro-Fp-3	CGGCTAGCCAATGTCTGTGTTAAAATTG	*BlVg* promoter	1899
Pro-Fp-4	CGGCTAGCGTAGATGTACTAGTTTTGTAG	*BlVg* promoter	1371
Pro-Fp-5	CTAGCTAGCGTTCACGGACATTTTCTTAG	*BlVg* promoter	1008
Pro-Fp-6	CTAGCTAGCATGTCCACGGGTTTCAACAT	*BlVg* promoter	919
Pro-rp	CCCAAGCTTATTCAAACTGACCAGTGTTC	*BlVg* promoter	
Pro-mut-FP	TGTCCCTGTAGTTTTAAGATTCACTGGAATATG	BrC-Z3 CRE mutation	
Pro-mut-RP	GTGAATCTTAAAACTACAGGGACAGTACATCTAC	BrC-Z3 CRE mutation	
VgS	GAAGAATCATCTGAGCAACGTG	*BlVg* qPCR	109
VgAS	GATGGTGCACTGTTTGCTTTTG		
β-actin-S	CCCGAGAGGAAATACTCTGT	Reference gene qPCR	104
β-actin-AS	GGTCCAGACTCGTCGTATTC		
BlBRC-Z-FP	CCTCGAGATGGGTTCGAGCCAACAGT	Protein expression	
BlBRC-Z1-RP	CGGTACCCTAAGGAATAGGACTAGATG		1491
BlBRC-Z2-RP	GGGTACCTTATTTAACATTAATACCATGAAC		1293
BlBRC-Z3-RP	GGGTACCTCACTTTCGAAAGTTCAATTTAGC		1257
BlBRC-Z4-RP	GGGTACCTTACGAGTCCAATTCGTTTTTCG		1239

### Bioinformatics analysis

Conserved domains were predicted by search the NCBI Conserved Domain Database [34]. The gene structure was displayed with GSDS (version 2.0) [35]. The potential transcription factor binding sites in the *BlVg* gene promoter were predicted by the MatInspector program (http://www.genomatix.de/). A phylogenetic tree based on the deduced amino acid sequences was constructed using Mega 7.0 software with the neighbor-joining method [36]. The reliability of branching was tested using bootstrap resampling with 1000 pseudoreplicates.

### Real-time quantitative PCR

The qRT-PCR data was normalized to the levels of the reference gene β-actin, and the amplification was performed on an Mx3000p qPCR system (Stratagene, Agilent, USA) with the SYBR Premix Ex Taq II kit (Takara, China) in a total volume of 20 μl. The thermocycling program was as follows: 95°C for 30 s, followed by 40 amplification cycles of 95°C for 5 s and 65°C for 30 s and a melting cycle from 65°C to 95°C. All biological samples were technically repeated in triplicate. Analysis of the qRT-PCR data was carried out using the 2^−ΔΔCt^ method of relative quantification [37]. The primers used are shown in Table 1.

### Cloning and plasmid construction of the 5'-flanking region of the *BlVg* gene

The 5'-flanking region of the *BlVg* gene was obtained using the Genome Walking Kit (Takara, China) with bumblebee genomic DNA as the template. Different lengths of the upstream regulatory region were amplified by PCR (with primers containing *NheI* and *HindIII* restriction sites) and cloned into the pGL4.11 [*luc2P*] vector (Promega, USA) for sequencing. The recombinant vectors were designated pGL4-Vg2.4K Luc, pGL4-Vg2.3K Luc, pGL4-Vg1.9K Luc, pGL4-Vg1.4K Luc, pGL4-Vg1K Luc, and pGL4-Vg0.9K Luc. All the plasmids were verified by automated sequencing analysis.

To analyze the functionality of the putative BrC-Z3 element within the *BlVg* promoter, specific mutations were introduced into pGL4-Vg1.9K Luc using overlap PCR. The core site of BrC-Z3 CRE, namely, “AAAC”, was changed to “GGGT”. The mutagenesis primers are shown in Table 1.

The open reading frames (ORFs) of *BlBrC-Z* (1–4) were amplified with whole body cDNA from an adult queen and cloned into the pBmFlag stable expression vector (stored in our laboratory; detailed information available in reference [38] with *XhoI* and *KpnI* sites for gene overexpression. The primers for the *BlVg* promoter, and *BlBrC-Z* (1–4) are shown in Table 1.

### Cell culture, transfection, and dual-luciferase assay

*Spodoptera frugiperda* 9 (*Sf9*) cell line was plated in 25-cm^2^ T-flasks (Corning) and maintained with TC-100 medium (Gibco) containing 10% heat-inactivated fetal bovine serum (FBS; Gibco) and streptomycin-penicillin solution (100 IU/mL penicillin and 100 mg/mL streptomycin, Amresco) at 27°C.

For transfection, cells were seeded in 96-well cell culture plates and transfected with pGL4-Vg Luc reporter and pRL-TK expression vector (the ratio of the two vectors (w/w) was 10: 1) using X-treme GENE HP DNA Transfection Reagent (Roche, USA) following the manufacturer’s instructions. For cotransfection of the BlBrC-Z protein (1–4), the ratio (w/w) of the reporter vector with the *BlVg* promoter, pBmFlag-BlBRC-Z (1–4) and the pRL-TK expression vector was 10:10:1. At 6 h after transfection, the culture medium was replaced with fresh medium containing FBS. After 24 h of transfection, the cells were treated with 0.2 μg/ml 20-hydroxyecdysone (20E) (Sigma, USA) or JH-III, which were dissolved in dimethyl sulfoxide (DMSO) at a stock concentration of 1 mg/ml and stored at -20°C. Controls were treated with 0.1% DMSO only. Cells were cultured for an additional 24 h and then lysed to detect luciferase activity with the Dual-Luciferase Reporter Assay Kit (Promega, USA) using a GloMax 20/20 Luminometer (Promega, USA) according to the manufacturer’s instructions. All assays were performed in triplicate.

### Western blotting

The cells transfected with the pBmFlag-BlBRC-Z (1–4) plasmid for 48 h were then lysed with RAPI buffer (Beyotime, China) containing one tablet of complete protease inhibitor (Roche, USA) per 100 ml at 4°C on a rocker for 30 min and then scraped. The recombinant proteins were electrophoresed on a 12% SDS-polyacrylamide gel, and then electrotransferred onto a PVDF membrane. After being washed for 5 min in PBST (pH 7.4), the membrane was blocked with blocking solution (5% skim milk, 17 ml of PBST) overnight at 4°C. Subsequently, the membrane was incubated with anti-Flag mouse monoclonal antibodies which were diluted 1:2000 with blocking solution at 37°C for 2 h. After washing three times with PBST (pH 7.4), the membrane was incubated in horseradish peroxidase (HRP) conjugated goat anti-mouse IgG secondary antibodies diluted 1:2000 with blocking solution at 37°C for 2 h. Finally, the membrane was washed three times with PBST, and the bands were detected by the DAB Horseradish Peroxidase Color Development Kit (Solarbio) according to the manufacturer’s instructions.

### Statistical analysis

The results were plotted using the Prism graphing program (GraphPad software, version 5.0, USA). The bars represent the mean ± SEM. The results were evaluated by one-way ANOVA with Tukey’s multiple comparison posthoc test (*P* < 0.05).

## Results

### Molecular characterization and phylogenetic analysis of the *B*. *lantschouensis* vitellogenin gene

**Fig 1:**
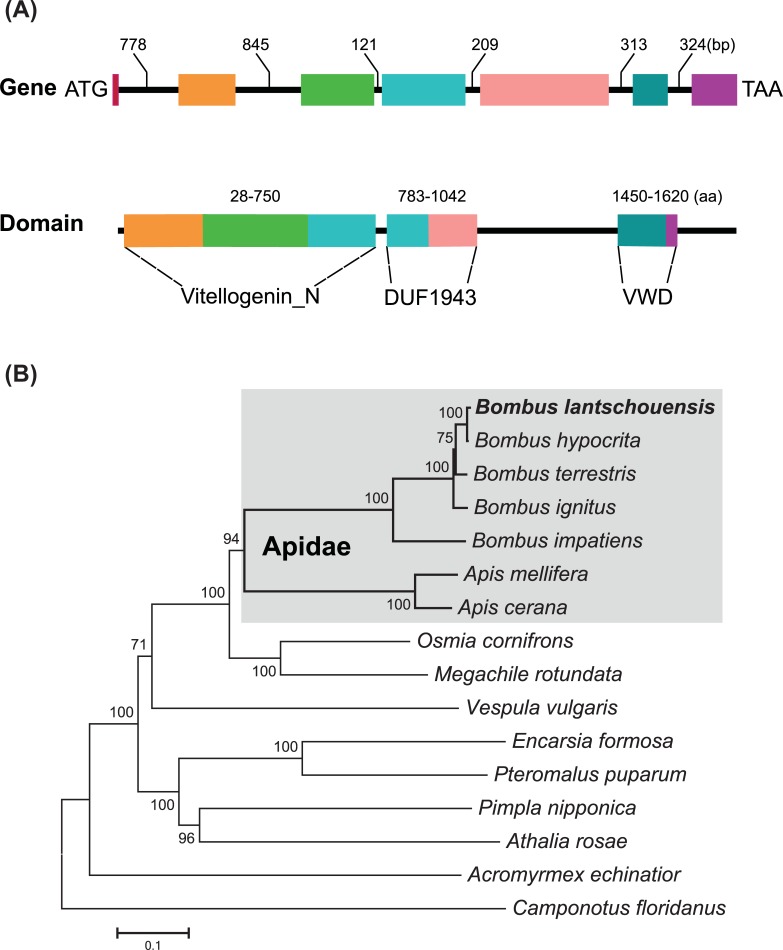
Gene structure, conserved domain and phylogenetic analysis of the *Vg* from *B*. *lantschouensis*. (A) Schematic representation of the *B*. *lantschouensis* vitellogenin (*BlVg*) gene and conserved protein domain. Exons are shown as rectangles with different colors; introns are represented by black lines, and the detailed length are shown. The protein BlVg domain was analyzed by a BLAST search of the NCBI Conserved Domain Database. (B) A phylogenetic tree was built based on hymenopteran Vg sequences by MEGA 7.0 using the Neighbor-Joining method with 1000 bootstrap values. Species from the family oApidae are highlighted in gray. The number on the nodes indicate the bootstrap values.

The full length ORF of *Vg* was amplified using RT-PCR with specific primers. The *B*. *lantschouensis vitellogenin* cDNA (GenBank accession no. MF632303) contained 5319 bp nucleotides, encoding a 1772-amino-acids protein with a theoretical isoelectric point (pI) of 6.21 and a calculated molecular weight (MW) of 201.5 kDa. The *BlVg* gene (GenBank accession no. MF632304) comprised seven exons and six introns, with a length of 7927 bp nucleotides (Fig 1A). The intron/exon rule (5’-donor GT…3’-acceptor AG) was conserved in the *BlVg* gene. The characteristic vitellogenin motifs are also shown in Fig 1A.

The similarity of BlVg with other hymenopteran Vg proteins is shown in S1 Table. The percentage of similarity ranged from 31 to 99%. The highest identity shared with *B*. *hypocrita* (99%), while the lowest similarity was observed with *Camponotus floridanus* Vg (31%).

Phylogenetic tree construction revealed that Vgs are evolutionarily conserved in Hymenoptera. In Apidae family, it is divided into two branches: honeybees and bumblebees, respectively. For the *Bombus* group, the BlVg protein is most closely related to *B*. *hypocrita* Vg, with which this protein formed a clade (Fig 1B).

### Expression profiles of *BlVg*

**Fig 2:**
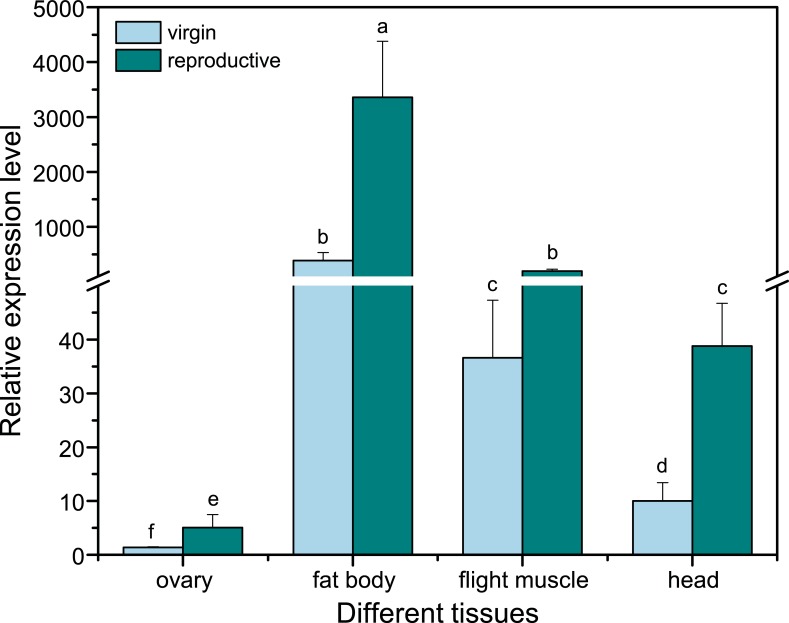
Analysis of relative *BlVg* expression in different tissues (ovary, fat body, flight muscle, head) of the bumblebee, *B*. *lantschouensis* under different reproductive statuses (virgin *vs* reproductive). The lowercase letters above the error bars show that the expression levels were significantly different (p<0.05) in the same tissue under different reproductive statuses.

To explore the spatial expression patterns of *BlVg* transcripts, various tissues, including head, flight muscle, fat body, and ovarian tissue, of *B*. *lantschouensis* queens were analyzed under different reproductive statuses (virgin vs reproductive). *BlVg* transcripts were expressed in all the tissues tested (Fig 2). As expected, the highest expression of *BlVg* transcripts was observed in the fat bodies (383.9-fold for virgin and 3357.07-fold for reproductive queens), followed by flight muscle, head, and ovaries (Fig 2). Additionally, reproductive queens exhibited a significantly higher level than virgins in each tissue (Fig 2).

### Regulation of *BlVg* by 20E and JH-III

To investigate the mechanism underlying the regulation of *BlVg* transcription, the 5’-upstream sequence of the *BlVg* gene from −1517 bp upstream of the translation start codon ATG to +895 bp of the *BlVg* intron (GenBank accession no. MF632305) was isolated using Genome Walking, and then analyzed using MatInspector software (http://www.genomatix.de/).

There were 89 CREs for transcription factor binding predicted on the *BlVg* promoter (S2 Table). For instance, the TATA box consensus sequence (ATAAA) was in close proximity upstream of the ATG translation start codon between −61 and −57. A conserved element (TTACTT) conforming to the arthropod promoter initiator (Inr) consensus sequence YYANTY was also located between −28 and −32 upstream of the ATG translation start codon. In the Inr conserved sequence, adenine is thought to be the transcription start site. Two CREs associated with ecdysone early-response genes were predicted to be DBRC-Z3 and DE74, respectively.

**Fig 3:**
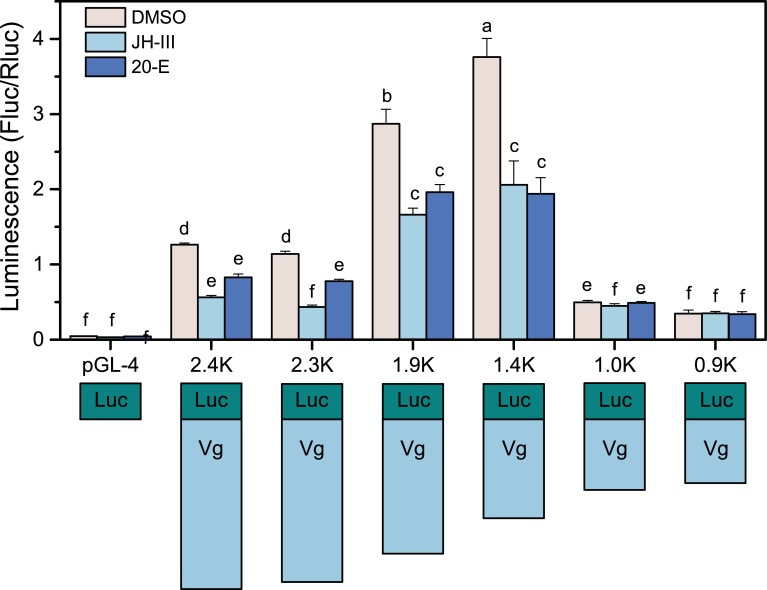
Effects of the hormonal solvent DMSO, 20E and JH-III on the activity of different lengths of *BlVg* promoter according to the luciferase activity measurement. The right panel indicates the reporter luciferase activity. The lowercase letters above the error bar show significant differences in luminescence (p<0.05). The left panel shows different lengths of the *BlVg* promoter cloned into the pGL-4.11 vector.

To determine whether activation of the *BlVg* promoter was enhanced by ecdysone or JH-III, a luciferase assay of promoter with different lengths was conducted in transfected *Sf9* cells. The promoter activity of distal 2.4 Kb, 2.3 Kb, and 1.9 Kb fragments, which contained the two putative ecdysone-related binding sites (DBRC-Z3 and DE74), was significantly enhanced by the addition of 20E but not JH-III (Fig 3). The promoter activity of the 1.4 Kb fragment harboring the DBRC-Z3 binding site was also considerably enhanced by only 20E (Fig 3). The promoter activities of both the Vg1.0K-Luc and Vg0.9K-Luc deletion clones, lacking ecdysone-related binding sites, did not change under ecdysone treatment. These results indicated that the regulation of *BlVg* transcription can be induced by ecdysone.

### Transcription factor BlBrC-Z3 enhanced *BlVg* promoter activity

**Fig 4:**
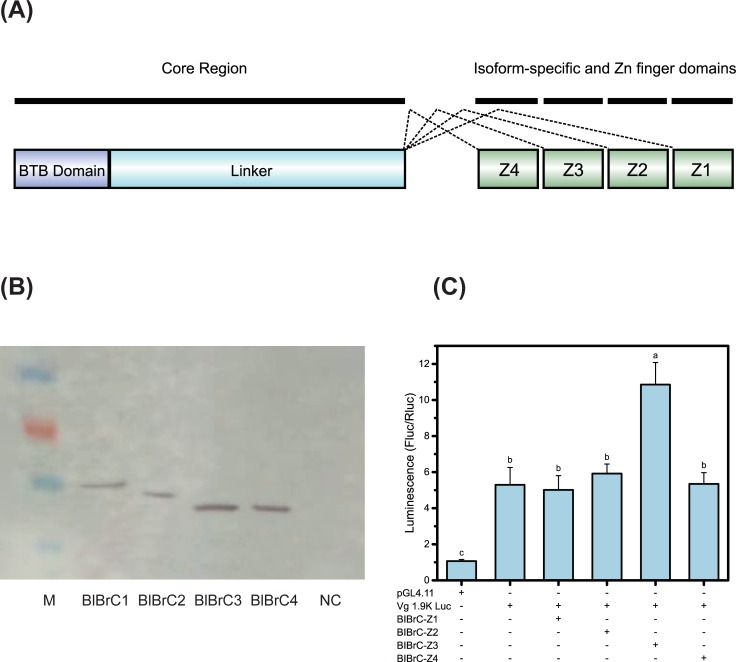
Functional characterization of BlBrC isoforms on the *BlVg* promoter activity. (A) Domain structures of BlBrC isoforms. The bent lines indicate the alternative splicing mode. (B) Western blot of recombinant BlBrC isoforms with anti-Flag antibodies. (C) *BlVg* promoter activity with coexpression of BlBrC isoforms in *Sf9* cell line.

To determine whether BlBrC-Z3 directly activated the transcription of *BlVg*, four alternatively spliced BrC isoforms (Z1, Z2, Z3 and Z4) were identified and the CDS domain structures of these isoforms are shown in Fig 4A. BlBR-C proteins have a common core region, including the BTB domain, and one of four isoform-specific C2H2 zinc finger domains (Z1, Z2, Z3, and Z4) generated by alternative splicing.

Luciferase assays were performed using *Sf9* cells co-transfected with pGL4-Vg1.9K Luc and pBmFlag-BlBrC isoforms (Z1, Z2, Z3 and Z4). Based on western-blot analysis, the four isoforms BlBrC-Z1, BlBrC-Z2, BlBrC-Z3 and BlBrC-Z4 were successfully expressed in the *Sf9* cell line (Fig 4B). The *BlVg* promoter activity was markedly increased only upon coexpression with the BlBrC-Z3 isoform (Fig 4C).

**Fig 5:**
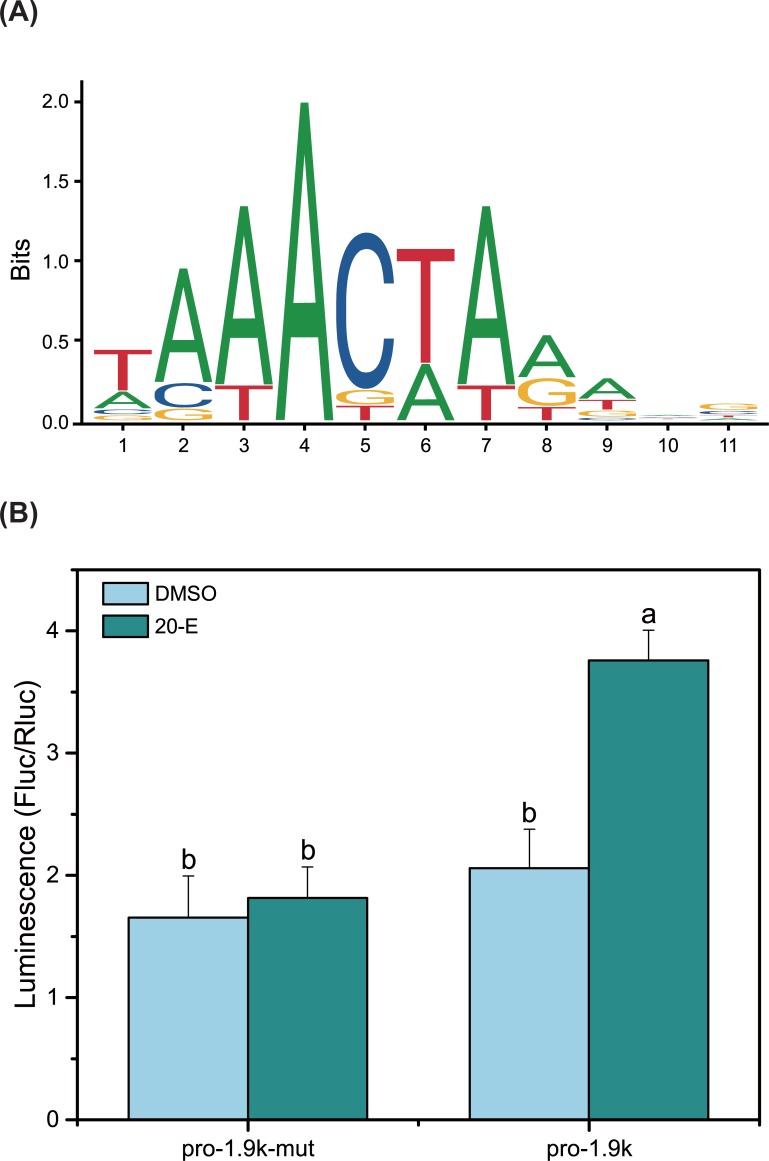
Characterization of the putative BlBrC-Z3 CRE and its effect on the *BlVg* promoter. (A) The putative conserved sequence of the BrC-Z3 response element predicted by JASPAR 5.0 ALPHA. Vertical axis indicated the frequency matrix. The numbers under the horizontal axis indicated the base position. (B) *BlVg* promoter activity with/without the BrC-Z3 CRE mutation under 20E treatment.

The putative conserved sequence of the BrC-Z3 CRE within the *BlVg* promoter is shown in Fig 5A. To characterize the function of the putative BrC-Z3 CRE on the *BlVg* promoter, reporter assays were performed in *Sf9* cells using the pGL4-Vg1.9K Luc vector with or without mutation. The *BlVg* promoter activity was strongly enhanced by 20E, while mutation in the BlBrC-Z3 CRE abolished this 20E-mediated activation (Fig 5B).

## Discussion

The Vg gene plays a crucial role in embryonic development, serving as the main source of nutrients for oocyte growth and development, and has been identified in many insects [1]. In this study, the genomic structure of the *Vg* from *B*. *lantschouensis* was identified and characterized. Analysis using the NCBI Conserved Domain Database showed thatBlVg contained all the typical vitellogenin domains, including Vitellogenin_N, DUF1943, and VWD at the C-terminus (Fig 1A). There was little amino acid difference between the protein from *B*. *lantschouensis* and those from other *Bombus* species (e.g. *B*. *ignitus*, *B*. *hypocrita*, and *B*. *terrestris*), which was consistent with the phylogenetic analysis (Fig 1B).

Vitellogenin is predominantly expressed in the fat bodies of insects [39] and mainly serves as storage for yolk protein. The fat body is considered as the main location of Vg synthesis [40]. The tissue expression profiles of *BlVg*, with ubiquitous expression observed in virtually all tissues and strongest expression observed in the abdominal fat bodies, were similar to those found in *B*. *terrestris* [14]. *BlVg* transcripts were found in a variety of tissues, including head and flight muscle, suggesting that these transcripts might have novel functions, such as anti-inflammatory and antioxidative defense functions, as observed in other honeybee species [41, 42].

The transcriptional regulation of insect *Vg* is a complex process that is determined by the promoter region, and requires multiple transcription factors. To elucidate the putative molecular mechanism of the transcriptional regulation of *BlVg*, a 2418-bp 5’-flanking region was isolated and many putative binding sites for transcription factors were predicted. These transcription factors, such as Drosophila heat shock factor (HSF), DE74A, DBrC-Z3, and C/EBP factors, are involved in development and in the response to environmental stress. In addition, numerous homeobox transcription factors, such as the Onecut transcription factor POU, were correlated with tissue segmentation [43].

Many studies have showed that *Vg* transcription is regulated by various transcription factors. For example, in the mosquito *A*. *aegypti*, *Vg* transcription is regulated by multiple transcription factors including the Forkhead box (Fox), GATA, BrC, E74, and E75 [31, 44–47]. In the silkworm *B*. *mori*, transcriptional regulation of *BmVg* is mediated by the binding of BmBrC-Z2 to the corresponding CRE in the *BmVg* promoter under the induction of 20E [32]. Lin et al verified that BmPOUM2 interacts with only BmBrC-Z2 to collaboratively regulate *BmVg* expression [33].

Ecdysone response transcription factors usually include the ecdysone receptor, the EcR/USP complex, and other early-response factors such as E74, E75, and BrC. For instance, the involvement of the EcR/USP complex in the induction of yolk protein genes has been elucidated in mosquito [48]. In addition, the mosquito *Vg* transcription is also regulated by E74 and E75 [49, 50]. In this study, only two early-response factors CREs E74 and BrC3 were predicted in the *BlVg* promoter. Interestingly, a putative BR-C was also identified in the promoter of the *Apis cerana cerana Vg* gene [51]. These predicted results indicated that the transcription of *BlVg* and *AcVg* might be regulated by the same transcription factor, namely, BrC.

BrC is an important ecdysone responsive early gene and plays a crucial role in the development and metamorphosis of insects [52]. In the present study, BlBrC isoforms (Z1, Z2, Z3, and Z4) were obtained and expressed successfully in *Sf9* cell line. The genomic BlBrC structure was different from that of the protein from *A*. *mellifera* L.. The order of the zinc-fingers within the BlBrC gene was Z4, Z3, Z2, and Z1, while in *A*. *mellifera* L., the order was Z1, Z4, Z2, and Z3 [25]. The arrangement of zinc-finger domain might be associated with protein function and warrants further study. In the transcriptional regulation of mosquito *Vg*, BrC-Z2, served as an enhancer, while Z1 and Z4 were repressors [31]. BmBrC-Z2 regulated *BmVg* transcription under the ecdysone signal [32]. In the present study, the reporter assays indicated that the transcription factor BlBrC-Z3 participates in the regulation of *BlVg* transcription by directly binding with the BrC-Z3 CRE under the induced ecdysone signal.

Other transcriptional factors could also be involved in transcriptional regulation of *Vg*. For instance, in the shrimp *Metapenaeus ensis*, Hsc70 acts as a molecular chaperone to negatively regulate *MeVg2* gene expression by binding to the HSF-response element of the *MeVg2* promoter [53].

These findings suggest that the transcriptional regulation of *Vg* is complex, and may be influenced by BrC, E74, the homeodomain and/or GATA transcription factors. Hence, the roles of the homeodomain and other transcription factors in the transcriptional regulation of *BlVg* needs to be further determined.

## Conclusion

In this study, we reported the molecular cloning and characterization of the *Vg* gene of the bumblebee *B*. *lantschouensis*. The *BlVg* genomic structure contained seven exons and six introns with a length of 7927 bp nucleotides. BlVg possessed all the typical vitellogenin domains, including Vitellogenin N, DUF1943, and VWD at the C-terminus. The *BlVg* transcript levels were higher in the fat bodies of queens than in other tissues, which was consistent with the results for other known bumblebee Vgs. Isoforms of the transcription factor BlBrC (Z1, Z2, Z3, and Z4) were obtained and expressed successfully in *Sf9* cell line. In addition, *BlVg* transcription was seen to be regulated by BlBrC-Z3 via binding to the BlBrC-Z3 response element within the *BlVg* promoter under 20E induction.

## Supporting information

S1 Table Sequence alignment of BlVg with other hymenopteran Vg amino acid sequences. (DOCX)

S2 Table Predicted response elements of the *BlVg* promoter. The A of the *BlVg* start codon (ATG) is at the +1 position.
(DOCX)
