# Supplementary material for: Broad-complex Z3 contributes to the ecdysone-mediated transcriptional regulation of the vitellogenin gene in Bombus lantschouensis
Source: PLoS One. 2018 Nov 15;13(11):e0207275. doi: 10.1371/journal.pone.0207275 (PMC6237364; doi:10.1371/journal.pone.0207275)
Supplement: S1 Table — (DOCX) [file pone.0207275.s001.docx]

**S1 Table**

Sequence alignment of BlVg with other selected Hymenoptera Vg amino acid sequences.

| **Species** | **Accession Number** | **Amino Acids** | **Identity (%)** |
| --- | --- | --- | --- |
| *Bombus terrestris* | XP_012163499 | 1779 | 96 |
| *Bombus hypocrita* | ACU00433 | 1772 | 99 |
| *Bombus ignitus* | ACQ91623 | 1772 | 95 |
| *Bombus impatiens* | XP_003492277 | 1772 | 79 |
| *Apis mellifera* | NP_001011578 | 1770 | 53 |
| *Apis cerana* | AJE68889 | 1770 | 52 |
| *Pimpla nipponica* | AAC32024 | 1807 | 37 |
| *Pteromalus puparum* | ABO70318 | 1803 | 35 |
| *Encarsia formosa* | AAT48601 | 1814 | 35 |
| *Athalia rosae* | NP_001295471 | 1872 | 33 |
| *Osmia cornifrons* | AIY25493 | 1783 | 52 |
| *Megachile rotundata* | XP_012139919 | 1770 | 51 |
| *Vespula vulgaris* | AER70365 | 1756 | 39 |
| *Acromyrmex echinatior* | XP_011061921 | 1655 | 35 |
| *Camponotus floridanus* | EFN64902 | 1820 | 31 |
| *Encarsia formosa* | AAT48601 | 1814 | 35 |
