# Supplementary material for: Broad-complex Z3 contributes to the ecdysone-mediated transcriptional regulation of the vitellogenin gene in Bombus lantschouensis
Source: PLoS One. 2018 Nov 15;13(11):e0207275. doi: 10.1371/journal.pone.0207275 (PMC6237364; doi:10.1371/journal.pone.0207275)
Supplement: S2 Table — The A of the BlVg start codon (ATG) is at the +1 position. (DOCX) [file pone.0207275.s002.docx]

**S2 Table**

The predicted response elements of *BlVg* promoter. The A of the *BlVg* start codon (ATG) is at the +1 position.

| **Gene** | **Detailed Information** | **DNA site** | **Link** | **Position** | | **Strand** | **Ration** | **Sequence** |
| --- | --- | --- | --- | --- | --- | --- | --- | --- |
|  |  |  |  | **from** | **to** |  |  |  |
| **Drosophila T-cell factor** | **Drosophila T-cell factor, homolog of TCF/LEF, interacts with Armadillo (b-Catenin homolog)** | **DTCF** | **DTCF.01** | **-1082** | **-1070** | **(+)** | **0.909** | **ttgtTTGAttatt** |
| **Drosophila homeobox transcription factor with CUT domain** | **Onecut transcription factor** | **DCUT** | **ONECUT.01** | **-1079** | **-1071** | **(+)** | **0.974** | **tttGATTat** |
| **Iroquois group of transcription factors** | **Mirror** | **IRXF** | **MIRR.01** | **-1041** | **-1033** | **(+)** | **0.997** | **aaaaaAACA** |
| **Drosophila segmentation gene tailless** | **Drosophila gap gene tailless, involved in embryonic segmentation** | **DTLL** | **TLL.01** | **-1029** | **-1021** | **(+)** | **0.965** | **acaagTAAA** |
| **Drosophila ecdysone induced protein E74A** | **Ecdysone-induced protein 74EF** | **DE74** | **EIP74EF.01** | **-1016** | **-1002** | **(+)** | **0.939** | **gatacaaGGAAgaaa** |
| **Drosophila segmentation gene tailless** | **Drosophila gap gene tailless, involved in embryonic segmentation** | **[DTLL](http://www.genomatix.de/cgi-bin/matinspector_prof/matrix_help.pl?s=cd85aa7da49005be4f83fb5beffd4c67;ML=94;NAME=FAM_I%24DTLL)** | **[TLL.01](http://www.genomatix.de/cgi-bin/matinspector_prof/matrix_help.pl?s=cd85aa7da49005be4f83fb5beffd4c67;ML=94;NAME=I%24TLL.01)** | **-1004** | **-996** | **(+)** | **0.969** | **aaaatTCAA** |
| **Paired homeodomain factors** | **Drosophila paired homeodomain** | **[PRDH](http://www.genomatix.de/cgi-bin/matinspector_prof/matrix_help.pl?s=cd85aa7da49005be4f83fb5beffd4c67;ML=94;NAME=FAM_I%24PRDH)** | **[PRD_HD.01](http://www.genomatix.de/cgi-bin/matinspector_prof/matrix_help.pl?s=cd85aa7da49005be4f83fb5beffd4c67;ML=94;NAME=I%24PRD_HD.01)** | **-998** | **-988** | **(+)** | **0.750** | **gttaaaattgttGATTatgac** |
| **Transcription factors with POU-domain - N-terminal to homeobox domain** | **Ventral veins lacking** | **[POUF](http://www.genomatix.de/cgi-bin/matinspector_prof/matrix_help.pl?s=cd85aa7da49005be4f83fb5beffd4c67;ML=94;NAME=FAM_I%24POUF)** | **[VVL.01](http://www.genomatix.de/cgi-bin/matinspector_prof/matrix_help.pl?s=cd85aa7da49005be4f83fb5beffd4c67;ML=94;NAME=I%24VVL.01)** | **-982** | **-970** | **(+)** | **0.960** | **attgttgATTAtg** |
| **Drosophila homeobox transcription factor with CUT domain** | **Onecut transcription factor** | **[DCUT](http://www.genomatix.de/cgi-bin/matinspector_prof/matrix_help.pl?s=cd85aa7da49005be4f83fb5beffd4c67;ML=94;NAME=FAM_I%24DCUT)** | **[ONECUT.01](http://www.genomatix.de/cgi-bin/matinspector_prof/matrix_help.pl?s=cd85aa7da49005be4f83fb5beffd4c67;ML=94;NAME=I%24ONECUT.01)** | **-979** | **-971** | **(+)** | **0.975** | **gttGATTat** |
| **Drosophila Abd-B group** | **Abdominal B** | **[ABDB](http://www.genomatix.de/cgi-bin/matinspector_prof/matrix_help.pl?s=cd85aa7da49005be4f83fb5beffd4c67;ML=94;NAME=FAM_I%24ABDB)** | **[ABDB.01](http://www.genomatix.de/cgi-bin/matinspector_prof/matrix_help.pl?s=cd85aa7da49005be4f83fb5beffd4c67;ML=94;NAME=I%24ABDB.01)** | **-967** | **-957** | **(+)** | **0.939** | **cgtcATAAatc** |
| **Drosophila Chorion Factor 2** | **CF2-II, Zinc finger splice variant II** | **[DCF2](http://www.genomatix.de/cgi-bin/matinspector_prof/matrix_help.pl?s=cd85aa7da49005be4f83fb5beffd4c67;ML=94;NAME=FAM_I%24DCF2)** | **[CF2II.02](http://www.genomatix.de/cgi-bin/matinspector_prof/matrix_help.pl?s=cd85aa7da49005be4f83fb5beffd4c67;ML=94;NAME=I%24CF2II.02)** | **-955** | **-947** | **(+)** | **0.881** | **gtAAATata** |
| **Drosophila Chorion Factor 2** | **CF2-II, Zinc finger splice variant II** | **[DCF2](http://www.genomatix.de/cgi-bin/matinspector_prof/matrix_help.pl?s=cd85aa7da49005be4f83fb5beffd4c67;ML=94;NAME=FAM_I%24DCF2)** | **[CF2II.01](http://www.genomatix.de/cgi-bin/matinspector_prof/matrix_help.pl?s=cd85aa7da49005be4f83fb5beffd4c67;ML=94;NAME=I%24CF2II.01)** | **-954** | **-946** | **(-)** | **0.933** | **tTATAttta** |
| **Drosophila homeoproteins** | **Antennapedia** | **[DHOM](http://www.genomatix.de/cgi-bin/matinspector_prof/matrix_help.pl?s=cd85aa7da49005be4f83fb5beffd4c67;ML=94;NAME=FAM_I%24DHOM)** | **[ANTP.01](http://www.genomatix.de/cgi-bin/matinspector_prof/matrix_help.pl?s=cd85aa7da49005be4f83fb5beffd4c67;ML=94;NAME=I%24ANTP.01)** | **-952** | **-936** | **(+)** | **0.952** | **aataTAATatgatgg** |
| **Vertebrate TATA binding protein factor** | **Lentivirus LTR TATA box** | **[VTBP](http://www.genomatix.de/cgi-bin/matinspector_prof/matrix_help.pl?s=cd85aa7da49005be4f83fb5beffd4c67;ML=94;NAME=FAM_O%24VTBP)** | **[LTATA.01](http://www.genomatix.de/cgi-bin/matinspector_prof/matrix_help.pl?s=cd85aa7da49005be4f83fb5beffd4c67;ML=94;NAME=O%24LTATA.01)** | **-953** | **-937** | **(+)** | **0.846** | **aaaTATAatatgatggc** |
| **Cis-acting silencer sequences binding Drosophila polycomp group proteins** | **PHO (pleiohomeotic) and PHO-like, polycomp group proteins (PcG) binding to PREs (polycomb group response elements)** | **[DPCG](http://www.genomatix.de/cgi-bin/matinspector_prof/matrix_help.pl?s=cd85aa7da49005be4f83fb5beffd4c67;ML=94;NAME=FAM_I%24DPCG)** | **[PHO.01](http://www.genomatix.de/cgi-bin/matinspector_prof/matrix_help.pl?s=cd85aa7da49005be4f83fb5beffd4c67;ML=94;NAME=I%24PHO.01)** | **-946** | **-932** | **(-)** | **0.893** | **tagatgCCATcatat** |
| **Drosophila Abd-B group** | **Abdominal B** | **[ABDB](http://www.genomatix.de/cgi-bin/matinspector_prof/matrix_help.pl?s=cd85aa7da49005be4f83fb5beffd4c67;ML=94;NAME=FAM_I%24ABDB)** | **[ABDB.01](http://www.genomatix.de/cgi-bin/matinspector_prof/matrix_help.pl?s=cd85aa7da49005be4f83fb5beffd4c67;ML=94;NAME=I%24ABDB.01)** | **-930** | **-920** | **(+)** | **0.950** | **catgATAAaaa** |
| **Drosophila Chorion Factor 1 /Ultraspiracle** | **Chorion factor 1, homologous to retinoid X receptor** | **[DCF1](http://www.genomatix.de/cgi-bin/matinspector_prof/matrix_help.pl?s=cd85aa7da49005be4f83fb5beffd4c67;ML=94;NAME=FAM_I%24DCF1)** | **[CF1.01](http://www.genomatix.de/cgi-bin/matinspector_prof/matrix_help.pl?s=cd85aa7da49005be4f83fb5beffd4c67;ML=94;NAME=I%24CF1.01)** | **-910** | **-902** | **(-)** | **1.000** | **gGGGTcacg** |
| **Drosophila homeoproteins** | **Extra-extra** | **[DHOM](http://www.genomatix.de/cgi-bin/matinspector_prof/matrix_help.pl?s=cd85aa7da49005be4f83fb5beffd4c67;ML=94;NAME=FAM_I%24DHOM)** | **[EXEX.01](http://www.genomatix.de/cgi-bin/matinspector_prof/matrix_help.pl?s=cd85aa7da49005be4f83fb5beffd4c67;ML=94;NAME=I%24EXEX.01)** | **-901** | **-887** | **(-)** | **0.875** | **gatgtgATTAcacgt** |
| **Drosphila giant transcription factor** | **Giant** | **[DGTF](http://www.genomatix.de/cgi-bin/matinspector_prof/matrix_help.pl?s=cd85aa7da49005be4f83fb5beffd4c67;ML=94;NAME=FAM_I%24DGTF)** | **[GT.01](http://www.genomatix.de/cgi-bin/matinspector_prof/matrix_help.pl?s=cd85aa7da49005be4f83fb5beffd4c67;ML=94;NAME=I%24GT.01)** | **-885** | **-871** | **(-)** | **0.917** | **taatatGTAAaatgc** |
| **Drosophila homeoproteins** | **BarH2** | **[DHOM](http://www.genomatix.de/cgi-bin/matinspector_prof/matrix_help.pl?s=cd85aa7da49005be4f83fb5beffd4c67;ML=94;NAME=FAM_I%24DHOM)** | **[BH2.01](http://www.genomatix.de/cgi-bin/matinspector_prof/matrix_help.pl?s=cd85aa7da49005be4f83fb5beffd4c67;ML=94;NAME=I%24BH2.01)** | **-862** | **-848** | **(-)** | **0.906** | **ttgaTAAAtgatgca** |
| **Paired homeodomain factors** | **Drosophila paired homeodomain** | **[PRDH](http://www.genomatix.de/cgi-bin/matinspector_prof/matrix_help.pl?s=cd85aa7da49005be4f83fb5beffd4c67;ML=94;NAME=FAM_I%24PRDH)** | **[PRD_HD.01](http://www.genomatix.de/cgi-bin/matinspector_prof/matrix_help.pl?s=cd85aa7da49005be4f83fb5beffd4c67;ML=94;NAME=I%24PRD_HD.01)** | **-865** | **-845** | **(-)** | **0.712** | **gtgttgataaatGATGcagtt** |
| **Vertebrate TATA binding protein factor** | **Muscle TATA box** | **[VTBP](http://www.genomatix.de/cgi-bin/matinspector_prof/matrix_help.pl?s=cd85aa7da49005be4f83fb5beffd4c67;ML=94;NAME=FAM_O%24VTBP)** | **[MTATA.01](http://www.genomatix.de/cgi-bin/matinspector_prof/matrix_help.pl?s=cd85aa7da49005be4f83fb5beffd4c67;ML=94;NAME=O%24MTATA.01)** | **-827** | **-811** | **(-)** | **0.933** | **cgataTAAAgccgtgtc** |
| **DNA replication-related element factor** | **DNA replication-related element factor** | **[DREF](http://www.genomatix.de/cgi-bin/matinspector_prof/matrix_help.pl?s=cd85aa7da49005be4f83fb5beffd4c67;ML=94;NAME=FAM_I%24DREF)** | **[DREF.01](http://www.genomatix.de/cgi-bin/matinspector_prof/matrix_help.pl?s=cd85aa7da49005be4f83fb5beffd4c67;ML=94;NAME=I%24DREF.01)** | **-818** | **-808** | **(-)** | **0.940** | **taaCGATataa** |
| **Boundary element associated factor** | **Boundary element-associated factor of 32kD** | **[BEAF](http://www.genomatix.de/cgi-bin/matinspector_prof/matrix_help.pl?s=cd85aa7da49005be4f83fb5beffd4c67;ML=94;NAME=FAM_I%24BEAF)** | **[BEAF32.01](http://www.genomatix.de/cgi-bin/matinspector_prof/matrix_help.pl?s=cd85aa7da49005be4f83fb5beffd4c67;ML=94;NAME=I%24BEAF32.01)** | **-817** | **-805** | **(-)** | **0.974** | **tattaaCGATata** |
| **DNA replication-related element factor** | **DNA replication-related element factor** | **[DREF](http://www.genomatix.de/cgi-bin/matinspector_prof/matrix_help.pl?s=cd85aa7da49005be4f83fb5beffd4c67;ML=94;NAME=FAM_I%24DREF)** | **[DREF.01](http://www.genomatix.de/cgi-bin/matinspector_prof/matrix_help.pl?s=cd85aa7da49005be4f83fb5beffd4c67;ML=94;NAME=I%24DREF.01)** | **-815** | **-805** | **(+)** | **0.839** | **tatCGTTaata** |
| **Drosophila homeoproteins** | **Extra-extra** | **[DHOM](http://www.genomatix.de/cgi-bin/matinspector_prof/matrix_help.pl?s=cd85aa7da49005be4f83fb5beffd4c67;ML=94;NAME=FAM_I%24DHOM)** | **[EXEX.01](http://www.genomatix.de/cgi-bin/matinspector_prof/matrix_help.pl?s=cd85aa7da49005be4f83fb5beffd4c67;ML=94;NAME=I%24EXEX.01)** | **-797** | **-783** | **(+)** | **0.875** | **actgtgATTAtgtgg** |
| **Drosophila Glia Cell Missing factors** | **Glial cells missing** | **[DGCM](http://www.genomatix.de/cgi-bin/matinspector_prof/matrix_help.pl?s=cd85aa7da49005be4f83fb5beffd4c67;ML=94;NAME=FAM_I%24DGCM)** | **[GCM.01](http://www.genomatix.de/cgi-bin/matinspector_prof/matrix_help.pl?s=cd85aa7da49005be4f83fb5beffd4c67;ML=94;NAME=I%24GCM.01)** | **-791** | **-779** | **(-)** | **0.945** | **ttacccACATaat** |
| **Drosophila supressor of Hairless** | **Suppressor of Hairless, linked to notch pathway** | **[DSUH](http://www.genomatix.de/cgi-bin/matinspector_prof/matrix_help.pl?s=cd85aa7da49005be4f83fb5beffd4c67;ML=94;NAME=FAM_I%24DSUH)** | **[SUH.01](http://www.genomatix.de/cgi-bin/matinspector_prof/matrix_help.pl?s=cd85aa7da49005be4f83fb5beffd4c67;ML=94;NAME=I%24SUH.01)** | **-789** | **-777** | **(+)** | **0.841** | **tatGTGGgtaact** |
| **Dead ringer factor** | **Dead ringer gene of Drosophila melanogaster** | **[DRIF](http://www.genomatix.de/cgi-bin/matinspector_prof/matrix_help.pl?s=cd85aa7da49005be4f83fb5beffd4c67;ML=94;NAME=FAM_I%24DRIF)** | **[DRI.01](http://www.genomatix.de/cgi-bin/matinspector_prof/matrix_help.pl?s=cd85aa7da49005be4f83fb5beffd4c67;ML=94;NAME=I%24DRI.01)** | **-777** | **-767** | **(-)** | **0.971** | **gagATTAaaaa** |
| **BTB/POZ proteins Bric a brac** | **Bric a brac 1** | **[BABF](http://www.genomatix.de/cgi-bin/matinspector_prof/matrix_help.pl?s=cd85aa7da49005be4f83fb5beffd4c67;ML=94;NAME=FAM_I%24BABF)** | **BAB1.01** | **-769** | **-753** | **(+)** | **0.877** | **ctcAATAaagatgttgg** |
| **Core promoter initiator elements** | **Drosophila initiator motifs** | **[INRE](http://www.genomatix.de/cgi-bin/matinspector_prof/matrix_help.pl?s=cd85aa7da49005be4f83fb5beffd4c67;ML=94;NAME=FAM_O%24INRE)** | **DINR.01** | **-740** | **-730** | **(+)** | **0.996** | **ttTCAGttgat** |
| **Paired homeodomain factors** | **Drosophila paired homeodomain** | **[PRDH](http://www.genomatix.de/cgi-bin/matinspector_prof/matrix_help.pl?s=cd85aa7da49005be4f83fb5beffd4c67;ML=94;NAME=FAM_I%24PRDH)** | **PRD_HD.01** | **-744** | **-724** | **(+)** | **0.734** | **ttattttcagttGATTaattt** |
| **Transcription factors with POU-domain - N-terminal to homeobox domain** | **Ventral veins lacking** | **[POUF](http://www.genomatix.de/cgi-bin/matinspector_prof/matrix_help.pl?s=cd85aa7da49005be4f83fb5beffd4c67;ML=94;NAME=FAM_I%24POUF)** | **[VVL.01](http://www.genomatix.de/cgi-bin/matinspector_prof/matrix_help.pl?s=cd85aa7da49005be4f83fb5beffd4c67;ML=94;NAME=I%24VVL.01)** | **-738** | **-726** | **(+)** | **0.963** | **tcagttgATTAat** |
| **Drosophila homeobox transcription factor with CUT domain** | **Onecut transcription factor** | **[DCUT](http://www.genomatix.de/cgi-bin/matinspector_prof/matrix_help.pl?s=cd85aa7da49005be4f83fb5beffd4c67;ML=94;NAME=FAM_I%24DCUT)** | **[ONECUT.01](http://www.genomatix.de/cgi-bin/matinspector_prof/matrix_help.pl?s=cd85aa7da49005be4f83fb5beffd4c67;ML=94;NAME=I%24ONECUT.01)** | **-735** | **-727** | **(+)** | **0.982** | **gttGATTaa** |
| **Drosophila homeoproteins** | **Deformed, homeotic gene in drosophila development** | **[DHOM](http://www.genomatix.de/cgi-bin/matinspector_prof/matrix_help.pl?s=cd85aa7da49005be4f83fb5beffd4c67;ML=94;NAME=FAM_I%24DHOM)** | **[DFD.01](http://www.genomatix.de/cgi-bin/matinspector_prof/matrix_help.pl?s=cd85aa7da49005be4f83fb5beffd4c67;ML=94;NAME=I%24DFD.01)** | **-738** | **-724** | **(-)** | **0.992** | **aaatTAATcaactga** |
| **Dead ringer factor** | **Dead ringer gene of Drosophila melanogaster** | **[DRIF](http://www.genomatix.de/cgi-bin/matinspector_prof/matrix_help.pl?s=cd85aa7da49005be4f83fb5beffd4c67;ML=94;NAME=FAM_I%24DRIF)** | **[DRI.01](http://www.genomatix.de/cgi-bin/matinspector_prof/matrix_help.pl?s=cd85aa7da49005be4f83fb5beffd4c67;ML=94;NAME=I%24DRI.01)** | **-734** | **-724** | **(+)** | **1.000** | **ttgATTAattt** |
| **Dead ringer factor** | **Dead ringer gene of Drosophila melanogaster** | **[DRIF](http://www.genomatix.de/cgi-bin/matinspector_prof/matrix_help.pl?s=cd85aa7da49005be4f83fb5beffd4c67;ML=94;NAME=FAM_I%24DRIF)** | **[DRI.01](http://www.genomatix.de/cgi-bin/matinspector_prof/matrix_help.pl?s=cd85aa7da49005be4f83fb5beffd4c67;ML=94;NAME=I%24DRI.01)** | **-733** | **-723** | **(-)** | **0.969** | **aaaATTAatca** |
| **Drosophila homeoproteins** | **Deformed, homeotic gene in drosophila development** | **[DHOM](http://www.genomatix.de/cgi-bin/matinspector_prof/matrix_help.pl?s=cd85aa7da49005be4f83fb5beffd4c67;ML=94;NAME=FAM_I%24DHOM)** | **[DFD.01](http://www.genomatix.de/cgi-bin/matinspector_prof/matrix_help.pl?s=cd85aa7da49005be4f83fb5beffd4c67;ML=94;NAME=I%24DFD.01)** | **-733** | **-719** | **(+)** | **0.990** | **tgatTAATtttcttc** |
| **Drosophila STAT** | **Signal transducers and activators of transcription** | **[DSTA](http://www.genomatix.de/cgi-bin/matinspector_prof/matrix_help.pl?s=cd85aa7da49005be4f83fb5beffd4c67;ML=94;NAME=FAM_I%24DSTA)** | **[STAT.01](http://www.genomatix.de/cgi-bin/matinspector_prof/matrix_help.pl?s=cd85aa7da49005be4f83fb5beffd4c67;ML=94;NAME=I%24STAT.01)** | **-727** | **-713** | **(-)** | **0.836** | **tccttcgaaGAAAat** |
| **Drosophila homeoproteins** | **Ultrabithorax** | **[DHOM](http://www.genomatix.de/cgi-bin/matinspector_prof/matrix_help.pl?s=cd85aa7da49005be4f83fb5beffd4c67;ML=94;NAME=FAM_I%24DHOM)** | **[UBX.01](http://www.genomatix.de/cgi-bin/matinspector_prof/matrix_help.pl?s=cd85aa7da49005be4f83fb5beffd4c67;ML=94;NAME=I%24UBX.01)** | **-700** | **-686** | **(+)** | **0.922** | **tttTTAAtttaaagg** |
| **Drosophila heat shock factors** | **Heat shock factor (Drosophila)** | **[DHSF](http://www.genomatix.de/cgi-bin/matinspector_prof/matrix_help.pl?s=cd85aa7da49005be4f83fb5beffd4c67;ML=94;NAME=FAM_I%24DHSF)** | **[HSF.01](http://www.genomatix.de/cgi-bin/matinspector_prof/matrix_help.pl?s=cd85aa7da49005be4f83fb5beffd4c67;ML=94;NAME=I%24HSF.01)** | **-688** | **-664** | **(-)** | **0.882** | **tgtcaGGAAttttctagaatgccct** |
| **Drosophila heat shock factors** | **Heat shock factor (Drosophila)** | **[DHSF](http://www.genomatix.de/cgi-bin/matinspector_prof/matrix_help.pl?s=cd85aa7da49005be4f83fb5beffd4c67;ML=94;NAME=FAM_I%24DHSF)** | **[HSF.01](http://www.genomatix.de/cgi-bin/matinspector_prof/matrix_help.pl?s=cd85aa7da49005be4f83fb5beffd4c67;ML=94;NAME=I%24HSF.01)** | **-683** | **-659** | **(+)** | **0.910** | **attctAGAAaattcctgacaggaat** |
| **Paired homeodomain factors** | **Drosophila paired homeodomain** | **[PRDH](http://www.genomatix.de/cgi-bin/matinspector_prof/matrix_help.pl?s=cd85aa7da49005be4f83fb5beffd4c67;ML=94;NAME=FAM_I%24PRDH)** | **[PRD_HD.01](http://www.genomatix.de/cgi-bin/matinspector_prof/matrix_help.pl?s=cd85aa7da49005be4f83fb5beffd4c67;ML=94;NAME=I%24PRD_HD.01)** | **-666** | **-646** | **(-)** | **0.714** | **tctattctaaagGATTcctgt** |
| **TGIF (TG-interacting factor)-Exd (extradenticle) group** | **Achintya** | **[TGIF](http://www.genomatix.de/cgi-bin/matinspector_prof/matrix_help.pl?s=cd85aa7da49005be4f83fb5beffd4c67;ML=94;NAME=FAM_I%24TGIF)** | **[ACHI.01](http://www.genomatix.de/cgi-bin/matinspector_prof/matrix_help.pl?s=cd85aa7da49005be4f83fb5beffd4c67;ML=94;NAME=I%24ACHI.01)** | **-582** | **-576** | **(+)** | **1.000** | **TGTCaaa** |
| **Boundary element associated factor** | **Boundary element-associated factor of 32kD** | **[BEAF](http://www.genomatix.de/cgi-bin/matinspector_prof/matrix_help.pl?s=cd85aa7da49005be4f83fb5beffd4c67;ML=94;NAME=FAM_I%24BEAF)** | **[BEAF32.01](http://www.genomatix.de/cgi-bin/matinspector_prof/matrix_help.pl?s=cd85aa7da49005be4f83fb5beffd4c67;ML=94;NAME=I%24BEAF32.01)** | **-562** | **-550** | **(+)** | **0.912** | **atagaaCGATaga** |
| **DNA replication-related element factor** | **DNA replication-related element factor** | **[DREF](http://www.genomatix.de/cgi-bin/matinspector_prof/matrix_help.pl?s=cd85aa7da49005be4f83fb5beffd4c67;ML=94;NAME=FAM_I%24DREF)** | **[DREF.01](http://www.genomatix.de/cgi-bin/matinspector_prof/matrix_help.pl?s=cd85aa7da49005be4f83fb5beffd4c67;ML=94;NAME=I%24DREF.01)** | **-559** | **-549** | **(+)** | **0.905** | **gaaCGATagaa** |
| **DNA replication-related element factor** | **DNA replication-related element factor** | **[DREF](http://www.genomatix.de/cgi-bin/matinspector_prof/matrix_help.pl?s=cd85aa7da49005be4f83fb5beffd4c67;ML=94;NAME=FAM_I%24DREF)** | **[DREF.01](http://www.genomatix.de/cgi-bin/matinspector_prof/matrix_help.pl?s=cd85aa7da49005be4f83fb5beffd4c67;ML=94;NAME=I%24DREF.01)** | **-534** | **-524** | **(-)** | **0.801** | **aatTGATagaa** |
| **Drosophila C/EBP like bZIP transcription factors** | **Slow border cells** | **[CEBP](http://www.genomatix.de/cgi-bin/matinspector_prof/matrix_help.pl?s=cd85aa7da49005be4f83fb5beffd4c67;ML=94;NAME=FAM_I%24CEBP)** | **[SLBO.01](http://www.genomatix.de/cgi-bin/matinspector_prof/matrix_help.pl?s=cd85aa7da49005be4f83fb5beffd4c67;ML=94;NAME=I%24SLBO.01)** | **-514** | **-502** | **(-)** | **0.924** | **tATTGaaaaagaa** |
| **Drosophila fork head factors** | **Crocodile regulator of head development, member of fork head domain protein family** | **[FKHD](http://www.genomatix.de/cgi-bin/matinspector_prof/matrix_help.pl?s=cd85aa7da49005be4f83fb5beffd4c67;ML=94;NAME=FAM_I%24FKHD)** | **[CROC.01](http://www.genomatix.de/cgi-bin/matinspector_prof/matrix_help.pl?s=cd85aa7da49005be4f83fb5beffd4c67;ML=94;NAME=I%24CROC.01)** | **-510** | **-494** | **(+)** | **0.993** | **ttttcaaTAAAtatctt** |
| **BTB/POZ proteins Bric a brac** | **Bric a brac 1** | **[BABF](http://www.genomatix.de/cgi-bin/matinspector_prof/matrix_help.pl?s=cd85aa7da49005be4f83fb5beffd4c67;ML=94;NAME=FAM_I%24BABF)** | **[BAB1.01](http://www.genomatix.de/cgi-bin/matinspector_prof/matrix_help.pl?s=cd85aa7da49005be4f83fb5beffd4c67;ML=94;NAME=I%24BAB1.01)** | **-508** | **-492** | **(+)** | **0.941** | **ttcAATAaatatcttta** |
| **BTB/POZ proteins Bric a brac** | **Bric a brac 1** | **[BABF](http://www.genomatix.de/cgi-bin/matinspector_prof/matrix_help.pl?s=cd85aa7da49005be4f83fb5beffd4c67;ML=94;NAME=FAM_I%24BABF)** | **[BAB1.01](http://www.genomatix.de/cgi-bin/matinspector_prof/matrix_help.pl?s=cd85aa7da49005be4f83fb5beffd4c67;ML=94;NAME=I%24BAB1.01)** | **-504** | **-488** | **(+)** | **0.882** | **ataAATAtctttatttt** |
| **BTB/POZ proteins Bric a brac** | **Bric a brac 1** | **[BABF](http://www.genomatix.de/cgi-bin/matinspector_prof/matrix_help.pl?s=cd85aa7da49005be4f83fb5beffd4c67;ML=94;NAME=FAM_I%24BABF)** | **[BAB1.01](http://www.genomatix.de/cgi-bin/matinspector_prof/matrix_help.pl?s=cd85aa7da49005be4f83fb5beffd4c67;ML=94;NAME=I%24BAB1.01)** | **-503** | **-487** | **(-)** | **0.966** | **aaaAATAaagatattta** |
| **Drosophila broad-complex for ecdysone steroid response** | **Broad-Complex Z3 Zinc Finger isoform** | **[DBRC](http://www.genomatix.de/cgi-bin/matinspector_prof/matrix_help.pl?s=cd85aa7da49005be4f83fb5beffd4c67;ML=94;NAME=FAM_I%24DBRC)3** | **BRCZ3.01** | **-482** | **-464** | **(-)** | **0.955** | **aactacaAAACtagtacat** |
| **Drosophila fork head factors** | **Crocodile regulator of head development, member of fork head domain protein family** | **[FKHD](http://www.genomatix.de/cgi-bin/matinspector_prof/matrix_help.pl?s=cd85aa7da49005be4f83fb5beffd4c67;ML=94;NAME=FAM_I%24FKHD)** | **[CROC.01](http://www.genomatix.de/cgi-bin/matinspector_prof/matrix_help.pl?s=cd85aa7da49005be4f83fb5beffd4c67;ML=94;NAME=I%24CROC.01)** | **-449** | **-433** | **(+)** | **0.984** | **aatatgaTAAAtattaa** |
| **Drosophila Chorion Factor 2** | **CF2-II, Zinc finger splice variant II** | **[DCF2](http://www.genomatix.de/cgi-bin/matinspector_prof/matrix_help.pl?s=cd85aa7da49005be4f83fb5beffd4c67;ML=94;NAME=FAM_I%24DCF2)** | **[CF2II.01](http://www.genomatix.de/cgi-bin/matinspector_prof/matrix_help.pl?s=cd85aa7da49005be4f83fb5beffd4c67;ML=94;NAME=I%24CF2II.01)** | **-425** | **-417** | **(+)** | **0.919** | **aTATAtttc** |
| **Drosphila giant transcription factor** | **Giant** | **[DGTF](http://www.genomatix.de/cgi-bin/matinspector_prof/matrix_help.pl?s=cd85aa7da49005be4f83fb5beffd4c67;ML=94;NAME=FAM_I%24DGTF)** | **[GT.01](http://www.genomatix.de/cgi-bin/matinspector_prof/matrix_help.pl?s=cd85aa7da49005be4f83fb5beffd4c67;ML=94;NAME=I%24GT.01)** | **-418** | **-404** | **(+)** | **0.919** | **tcatatGTCAcaacc** |
| **TGIF (TG-interacting factor)-Exd (extradenticle) group** | **Homothorax** | **[TGIF](http://www.genomatix.de/cgi-bin/matinspector_prof/matrix_help.pl?s=cd85aa7da49005be4f83fb5beffd4c67;ML=94;NAME=FAM_I%24TGIF)** | **[HTH.01](http://www.genomatix.de/cgi-bin/matinspector_prof/matrix_help.pl?s=cd85aa7da49005be4f83fb5beffd4c67;ML=94;NAME=I%24HTH.01)** | **-413** | **-407** | **(+)** | **1.000** | **tGTCAca** |
| **Drosophila Abd-B group** | **Abdominal B** | **[ABDB](http://www.genomatix.de/cgi-bin/matinspector_prof/matrix_help.pl?s=cd85aa7da49005be4f83fb5beffd4c67;ML=94;NAME=FAM_I%24ABDB)** | **[ABDB.01](http://www.genomatix.de/cgi-bin/matinspector_prof/matrix_help.pl?s=cd85aa7da49005be4f83fb5beffd4c67;ML=94;NAME=I%24ABDB.01)** | **-384** | **-374** | **(-)** | **0.923** | **tttcATAAagc** |
| **Core promoter initiator elements** | **Drosophila initiator motifs** | **[INRE](http://www.genomatix.de/cgi-bin/matinspector_prof/matrix_help.pl?s=cd85aa7da49005be4f83fb5beffd4c67;ML=94;NAME=FAM_O%24INRE)** | **[DINR.01](http://www.genomatix.de/cgi-bin/matinspector_prof/matrix_help.pl?s=cd85aa7da49005be4f83fb5beffd4c67;ML=94;NAME=O%24DINR.01)** | **-379** | **-369** | **(-)** | **0.954** | **taTCATttcat** |
| **Drosophila Abd-B group** | **Abdominal B** | **[ABDB](http://www.genomatix.de/cgi-bin/matinspector_prof/matrix_help.pl?s=cd85aa7da49005be4f83fb5beffd4c67;ML=94;NAME=FAM_I%24ABDB)** | **[ABDB.01](http://www.genomatix.de/cgi-bin/matinspector_prof/matrix_help.pl?s=cd85aa7da49005be4f83fb5beffd4c67;ML=94;NAME=I%24ABDB.01)** | **-375** | **-365** | **(+)** | **0.920** | **aatgATAAatc** |
| **Drosophila homeobox transcription factor with CUT domain** | **Onecut transcription factor** | **[DCUT](http://www.genomatix.de/cgi-bin/matinspector_prof/matrix_help.pl?s=cd85aa7da49005be4f83fb5beffd4c67;ML=94;NAME=FAM_I%24DCUT)** | **[ONECUT.01](http://www.genomatix.de/cgi-bin/matinspector_prof/matrix_help.pl?s=cd85aa7da49005be4f83fb5beffd4c67;ML=94;NAME=I%24ONECUT.01)** | **-370** | **-362** | **(-)** | **0.932** | **gctGATTta** |
| **Boundary element associated factor** | **Boundary element-associated factor of 32kD** | **BEAF** | **[BEAF32.01](http://www.genomatix.de/cgi-bin/matinspector_prof/matrix_help.pl?s=cd85aa7da49005be4f83fb5beffd4c67;ML=94;NAME=I%24BEAF32.01)** | **-353** | **-341** | **(-)** | **0.881** | **aattctCGATtta** |
| **DNA replication-related element factor** | **DNA replication-related element factor** | **DREF** | **[DREF.01](http://www.genomatix.de/cgi-bin/matinspector_prof/matrix_help.pl?s=cd85aa7da49005be4f83fb5beffd4c67;ML=94;NAME=I%24DREF.01)** | **-351** | **-341** | **(+)** | **0.809** | **aatCGAGaatt** |
| **Drosophila STAT** | **Signal transducers and activators of transcription** | **DSTA** | **[STAT.01](http://www.genomatix.de/cgi-bin/matinspector_prof/matrix_help.pl?s=cd85aa7da49005be4f83fb5beffd4c67;ML=94;NAME=I%24STAT.01)** | **-344** | **-330** | **(+)** | **0.928** | **aatttcgaaGAAAaa** |
| **Drosophila STAT** | **Signal transducers and activators of transcription** | **DSTA** | **[STAT.01](http://www.genomatix.de/cgi-bin/matinspector_prof/matrix_help.pl?s=cd85aa7da49005be4f83fb5beffd4c67;ML=94;NAME=I%24STAT.01)** | **-344** | **-330** | **(-)** | **0.931** | **tttttcttcGAAAtt** |
| **TGIF (TG-interacting factor)-Exd (extradenticle) group** | **Achintya** | **TGIF** | **[ACHI.01](http://www.genomatix.de/cgi-bin/matinspector_prof/matrix_help.pl?s=cd85aa7da49005be4f83fb5beffd4c67;ML=94;NAME=I%24ACHI.01)** | **-326** | **-320** | **(+)** | **1.000** | **TGTCaaa** |
| **Drosophila Chorion Factor 2** | **CF2-II, Zinc finger splice variant II** | **[DCF2](http://www.genomatix.de/cgi-bin/matinspector_prof/matrix_help.pl?s=cd85aa7da49005be4f83fb5beffd4c67;ML=94;NAME=FAM_I%24DCF2)** | **[CF2II.01](http://www.genomatix.de/cgi-bin/matinspector_prof/matrix_help.pl?s=cd85aa7da49005be4f83fb5beffd4c67;ML=94;NAME=I%24CF2II.01)** | **-309** | **-301** | **(+)** | **0.928** | **gTATAtttc** |
| **Drosphila giant transcription factor** | **Giant** | **[DGTF](http://www.genomatix.de/cgi-bin/matinspector_prof/matrix_help.pl?s=cd85aa7da49005be4f83fb5beffd4c67;ML=94;NAME=FAM_I%24DGTF)** | **[GT.01](http://www.genomatix.de/cgi-bin/matinspector_prof/matrix_help.pl?s=cd85aa7da49005be4f83fb5beffd4c67;ML=94;NAME=I%24GT.01)** | **-306** | **-292** | **(+)** | **0.925** | **tatttcGTTAcaact** |
| **Paired homeodomain factors** | **Drosophila paired homeodomain** | **[PRDH](http://www.genomatix.de/cgi-bin/matinspector_prof/matrix_help.pl?s=cd85aa7da49005be4f83fb5beffd4c67;ML=94;NAME=FAM_I%24PRDH)** | **[PRD_HD.01](http://www.genomatix.de/cgi-bin/matinspector_prof/matrix_help.pl?s=cd85aa7da49005be4f83fb5beffd4c67;ML=94;NAME=I%24PRD_HD.01)** | **-285** | **-265** | **(+)** | **0.708** | **aacgaggaaaagGATTagttc** |
| **Bicoid-like homeodomain transcription factors** | **K50 type homeodomain site selected with drosophila orthodenticle (OTD) homeodomain** | **BCDF** | **[K50_HD.01](http://www.genomatix.de/cgi-bin/matinspector_prof/matrix_help.pl?s=cd85aa7da49005be4f83fb5beffd4c67;ML=94;NAME=I%24K50_HD.01)** | **-278** | **-266** | **(-)** | **0.960** | **aacTAATcctttt** |
| **Drosophila gap gene Krueppel** | **Krueppel, zinc finger protein, involved in body segmentation of the drosophila embryo** | **DKRU** | **KR.01** | **-277** | **-267** | **(+)** | **0.931** | **aaaGGATtagt** |
| **DNA replication-related element factor** | **DNA replication-related element factor** | **DREF** | **DREF.01** | **-261** | **-251** | **(-)** | **0.807** | **tatCGATttct** |
| **Boundary element associated factor** | **Boundary element-associated factor of 32kD** | **BEAF** | **BEAF32.01** | **-261** | **-249** | **(+)** | **0.907** | **agaaatCGATatc** |
| **DNA replication-related element factor** | **DNA replication-related element factor** | **DREF** | **DREF.01** | **-258** | **-248** | **(+)** | **0.948** | **aatCGATatcg** |
| **DNA replication-related element factor** | **DNA replication-related element factor** | **DREF** | **DREF.01** | **-255** | **-245** | **(-)** | **0.891** | **caaCGATatcg** |
| **Boundary element associated factor** | **Boundary element-associated factor of 32kD** | **[BEAF](http://www.genomatix.de/cgi-bin/matinspector_prof/matrix_help.pl?s=cd85aa7da49005be4f83fb5beffd4c67;ML=94;NAME=FAM_I%24BEAF)** | **BEAF32.01** | **-254** | **-242** | **(-)** | **0.880** | **cgtcaaCGATatc** |
| **Drosophila Abd-B group** | **Abdominal B** | **ABDB** | **ABDB.01** | **-206** | **-196** | **(+)** | **0.960** | **gataATAAaat** |
| **Drosophila homeoproteins** | **Antennapedia** | **[DHOM](http://www.genomatix.de/cgi-bin/matinspector_prof/matrix_help.pl?s=cd85aa7da49005be4f83fb5beffd4c67;ML=94;NAME=FAM_I%24DHOM)** | **ANTP.01** | **-208** | **-194** | **(+)** | **0.959** | **cagaTAATaaaattt** |
| **Transcription factors with POU-domain - N-terminal to homeobox domain** | **Ventral veins lacking** | **POUF** | **VVL.01** | **-206** | **-194** | **(-)** | **0.943** | **aaattttATTAtc** |
| **Paired homeodomain factors** | **Drosophila PAX6 P3 homeodomain binding site** | **PRDH** | **PAX6_HD.01** | **-210** | **-190** | **(+)** | **0.802** | **agcagataataaaATTTgacc** |
| **Paired homeodomain factors** | **Drosophila PAX6 P3 homeodomain binding site** | **PRDH** | **PAX6_HD.01** | **-208** | **-188** | **(-)** | **0.791** | **caggtcaaattttATTAtctg** |
| **Drosophila homeoproteins** | **Extra-extra** | **DHOM** | **EXEX.01** | **-187** | **-173** | **(+)** | **0.872** | **gtagttATTAtttta** |
| **Drosophila homeoproteins** | **Brain-specific homeobox** | **DHOM** | **BSH.01** | **-178** | **-164** | **(+)** | **0.979** | **atttTAATagacata** |
| **Transcription factors with POU-domain - N-terminal to homeobox domain** | **Ventral veins lacking** | **POUF** | **VVL.01** | **-126** | **-114** | **(+)** | **0.931** | **cgtgtagATTAaa** |
| **Dead ringer factor** | **Dead ringer gene of Drosophila melanogaster** | **DRIF** | **DRI.01** | **-122** | **-112** | **(+)** | **0.971** | **tagATTAaaaa** |
| **Drosophila homeoproteins** | **Brain-specific homeobox** | **DHOM** | **BSH.01** | **-77** | **-63** | **(-)** | **0.986** | **acttTAATcgtgcca** |
| **Dead ringer factor** | **Dead ringer gene of Drosophila melanogaster** | **DRIF** | **DRI.01** | **-73** | **-63** | **(+)** | **0.980** | **acgATTAaagt** |
| **Plant TATA binding protein factor** | **Plant TATA box** | **PTBP** | **PTATA.02** | **-67** | **-53** | **(+)** | **0.902** | **aaagTATAaattgga** |
| **Vertebrate TATA binding protein factor** | **Cellular and viral TATA box elements** | **VTBP** | **VTATA.01** | **-66** | **-50** | **(+)** | **0.956** | **aagtaTAAAttggaggt** |
